# PNI as a predictive biomarker: a novel nomogram of immunotherapy efficacy in advanced breast cancer

**DOI:** 10.3389/fonc.2025.1534545

**Published:** 2025-08-15

**Authors:** Xinyang Li, Xiang Cheng, Yikai Han, Xiaodan Liu, Yujin Fang, Shengju Ren, Xiangwen Dong, Ziwen Lei, Yue Zhang, Tengfei Zhang

**Affiliations:** ^1^ The First Affiliated Hospital of Henan University of Chinese Medicine, Zhengzhou, Henan, China; ^2^ Department of Oncology, The First Affiliated Hospital of Zhengzhou University, Zhengzhou, Henan, China; ^3^ Department of Oncology, Dengzhou Central Hospital, Dengzhou, Henan, China; ^4^ Medical School, Huanghe Science and Technology University, Zhengzhou, Henan, China

**Keywords:** breast cancer, nomogram, prognostic nutritional index, immunotherapy, prognosis

## Abstract

**Purpose:**

There has been a persistent upward trend in breast cancer (BC) incidence in recent years. The advancement of immunotherapy has introduced promising therapeutic options. This study focuses on identify potential biomarkers to predict clinical outcomes in advanced BC patients receiving immunotherapy.

**Patients and methods:**

In accordance with the predefined inclusion and exclusion criteria, a cohort of 154 patients were enrolled in this study. Progression-free survival (PFS) and overall survival (OS) were the primary endpoints. The end of follow-up is October 2024. Statistical analyses were performed utilizing IBM SPSS Statistics, version 26.0, and R software, version 4.3.1.

**Results:**

Univariate Cox regression analysis demonstrated a statistically significant association between the prognostic nutritional index (PNI) and both PFS and OS (p<0.05). Kaplan–Meier survival analysis, complemented by log-rank tests, revealed statistically differences in survival outcomes stratified by PNI levels (p<0.05). After adjusting for potential confounders in multivariate Cox regression analysis, PNI remained an independent prognostic factor in advanced BC patients undergoing immunotherapy. The predictive accuracy of the nomograms, as measured by the concordance indices (C-indices), was 0.710 for PFS and 0.705 for OS. The area under the ROC (AUC) for the predicted model at 6-, 12-, 18- and 24- months were 0.756, 0.761, 0.684, and 0.779. For OS, the AUC values were 0.753, 0.722, 0.641 and 0.576. The calibration curves revealed good concordance between the observed outcomes and the predicted probabilities.

**Conclusions:**

PNI is an independent prognostic factor for advanced BC receiving immunotherapy and the prognostic model based on PNI has good discrimination, authenticity and consistency.

## 1 Introduction

Breast cancer (BC) is one of the most prevalent malignant tumors in women (1). Current treatments include surgery, chemotherapy, radiotherapy, targeted therapy, and immunotherapy (2). While chemotherapy remains the primary treatment for advanced BC, the high rates of drug resistance limit its effectiveness in many patients (3). The emergence of immunotherapy as a promising treatment modality has spurred increasing interest in the potential synergistic effects of combining chemotherapy with immunotherapy across various cancer types (4), with many studies linking this combination therapy to better outcomes in BC and other malignancies (5–8).

A variety of markers have been identified that can predict the efficacy of immunotherapy in breast cancer, such as programmed cell death-ligand 1 (PD-L1) expression level (9), tumor mutation burden (TMB) (10), microsatellite instability-high (MSI-H) (11), and defective mismatch repair (dMMR) (11). Additionally, gene mutations such as those in EGFR (12), VEGF (13), and genes related to immunotherapy outcomes (e.g., Tp53, Ras, WDR4, and STK11) (14–17), may serve as potential predictive markers for the effectiveness of BC immunotherapy. Nevertheless, these biomarkers are not easily accessible, as they require the acquisition of sufficient tumor tissue for immunohistochemistry and genetic testing, and cannot fully predict the efficacy of immunotherapy.

Inflammation and nutritional indicators have made significant progress in predicting cancer prognosis (18). Inflammation-nutrition indicators primarily include cellular components such as neutrophils, lymphocytes, and monocytes, along with proteins albumin (ALB) and C-reactive protein (CRP). Additionally, these indicators encompass derived ratios, such as the neutrophil-to-lymphocyte ratio (NLR), monocyte-to-lymphocyte Ratio (MLR), platelet-to-lymphocyte ratio (PLR), systemic immune-inflammatory index (SII), and prognostic nutritional index (PNI). These markers collectively provide insights into the balance between inflammatory responses and nutritional status, offering valuable prognostic and diagnostic information in various clinical contexts. PNI is calculated by combining the ALB (g/dL) with five times the peripheral blood lymphocyte count (10^9/L), using the formula: PNI = ALB (g/dL) + 5 × (lymphocyte count in 10^9/L). This index is a widely used biomarker that reflects both nutritional status and immune competence, providing valuable prognostic information in various clinical settings, including oncology, surgery, and chronic disease management, such as gastric cancer (19), renal cancer (20), lung cancer (21), etc. In the above studies, interfering factors such as underlying diseases were excluded. This research was designed to investigate the potential of the PNI as a prognostic biomarker in advanced BC receiving immunotherapy. Compared with similar studies, this study integrates information on the clinical characteristics, immunohistochemistry status and composite inflammatory indicators to achieve combined modeling of multidimensional immunometabolism microenvironment and metastatic heterogeneity, providing a novel and clinically applicable tool for prognostic assessment in advanced BC patients treated with immunotherapy.

## 2 Materials and methods

### 2.1 Patients

Following a retrospective analysis of patients diagnosed with advanced BC and received immunotherapy at the First Affiliated Hospital of Zhengzhou University between January 2022 and May 2024, the specific immunotherapy refers to PD-1/PD-L1 inhibitors, a total of 154 patients were enrolled based on predefined inclusion and exclusion criteria (**Supplementary Figure S1**). Patients eligible for inclusion had to meet the following criteria: 1) a histologically confirmed diagnosis of BC, and the disease was locally advanced, recurrent, or metastatic, 2) female patients aged 18 years or older, 3) completion of a minimum of two cycles of immunotherapy, 4) the presence of evaluable lesions, and 5) regular follow-up with imaging studies and hematological examinations. The primary exclusion criteria of this study included: 1) combined with other tumors, 2) long-term use of hormones, immunosuppressants, or trauma or infection within 2 weeks before treatment that may affect the test results, 3) Patients with severe diseases of other organs, or 4) incomplete follow-up data. This study complies with the Declaration of Helsinki and was approved by the Ethics Committee of the First Affiliated Hospital of Zhengzhou University (Approval No. 2024-KY-1748-001). Written informed consent was obtained from all participating patients prior to their inclusion in the study.

### 2.2 Data collection

Several indicators were selected for analysis, including age, ER, PR, Her2 status, presence or absence of bone, lung, liver and brain metastasis, combination therapy, and multiple laboratory parameters. Hematological indices, such as platelet count, neutrophil count, lymphocyte count, monocyte count, and ALB levels, were retrospectively collected within one week before the first immunotherapy. Based on these data, composite hematological indices NLR, PLR, MLR, and PNI were calculated. PFS was used to evaluate the progression time of patients from the date of initiation of immunotherapy to recurrence or progression. OS was measured from the start of immunotherapy until death or the last follow-up. Treatment efficacy was assessed every two months using computed tomography (CT) and magnetic resonance imaging (MRI), in accordance with the Response Evaluation Criteria in Solid Tumors (RECIST version 1.1) guidelines.

### 2.3 Statistical analysis

The optimal cutoff values for continuous numerical variables were determined using X-tile (version 3.6.1). Statistical analyses, including descriptive statistics, Cox regression analysis, Kaplan-Meier analysis, and the Log-rank test, were performed using SPSS (version 26.0). Survival curves for each index were plotted using GraphPad Prism (version 8.0.2). Additionally, R software (version 4.3.1) was utilized to construct nomograms and verify its performance through internal verification (Bootstrap self-sampling method), including calculating the concordance index (C-index), generating receiver operating characteristic (ROC) curves, calibration curves, and decision curve analysis (DCA). The ROC curves were created using the *timeROC* package, calibration curves were generated with the *rms* package, and DCA was performed using the *ggDCA* package.

## 3 Results

### 3.1 Patient characteristics

According to the inclusion and exclusion criteria, 154 patients were enrolled in this study. The median OS was 346.50 days (range: 80.00-948.00 days), and the median PFS was 227.50 days (range: 25.00-862.00 days). At the end of follow-up, 65 (42.2%) patients had the following outcome event: death. The median age of the study population was 52 years (range: 31–71 years). On the whole, 53 patients (34.4%) had bone metastases, 39 (25.3%) had lung metastases, 33 (21.4%) had liver metastases, and 11 (7.1%) had brain metastases. Additional clinical characteristics are summarized in **Table 1**.

**Table 1:** Clinical characteristics of patients.

Characteristics	n=154
Age (median, range), years	52.00 (31.00–71.00)
ER (Negative/Positive)	98 (63.6%)/56 (36.4%)
PR (Negative/Positive)	109 (70.8%)/45 (29.2%)
Her2 (Negative/Positive)	140 (90.9%)/14 (9.1%)
Bone Metastasis (without/with)	101 (65.6%)/53 (34.4%)
Lung Metastasis (without/with)	115 (74.7%)/39 (25.3%)
Liver Metastasis (without/with)	121 (78.6%)/33 (21.4%)
Brain Metastasis (without/with)	143 (92.9%)/11 (7.1%)
Combination therapy (without/with)	4 (2.6%)/150 (97.4%)
PFS (median, range), days	227.50 (25.00-862.00)
OS (median, range), days	346.50 (80.00-948.00))
Status (alive/dead)	89 (57.8%)/65 (42.2%)
NLR (median, range)	2.59 (0.36-16.35)
PLR (median, range)	187.81 (61.17-885.00)
MLR (median, range)	0.30 (0.06-1.76))
PNI (median, range)	47.73 (31.90–59.70)

### 3.2 Acquisition of the optimal cutoff value

X-tile software is a purpose to evaluate the biological relationship between biomarkers and certain disease outcomes, which can include time factors and generate corrected P values to evaluate the statistical significance between data truncated by multiple cutoff points. PNI, calculated as the sum of five times the peripheral blood lymphocyte counts and serum ALB level, was analyzed using X-tile software to determine the optimal cutoff value. Based on this value, patients were stratified into two groups: a low-level group (PNI < 47.50) and a high-level group (PNI ≥ 47.50).

### 3.3 Survival analysis

**Figure 1:**
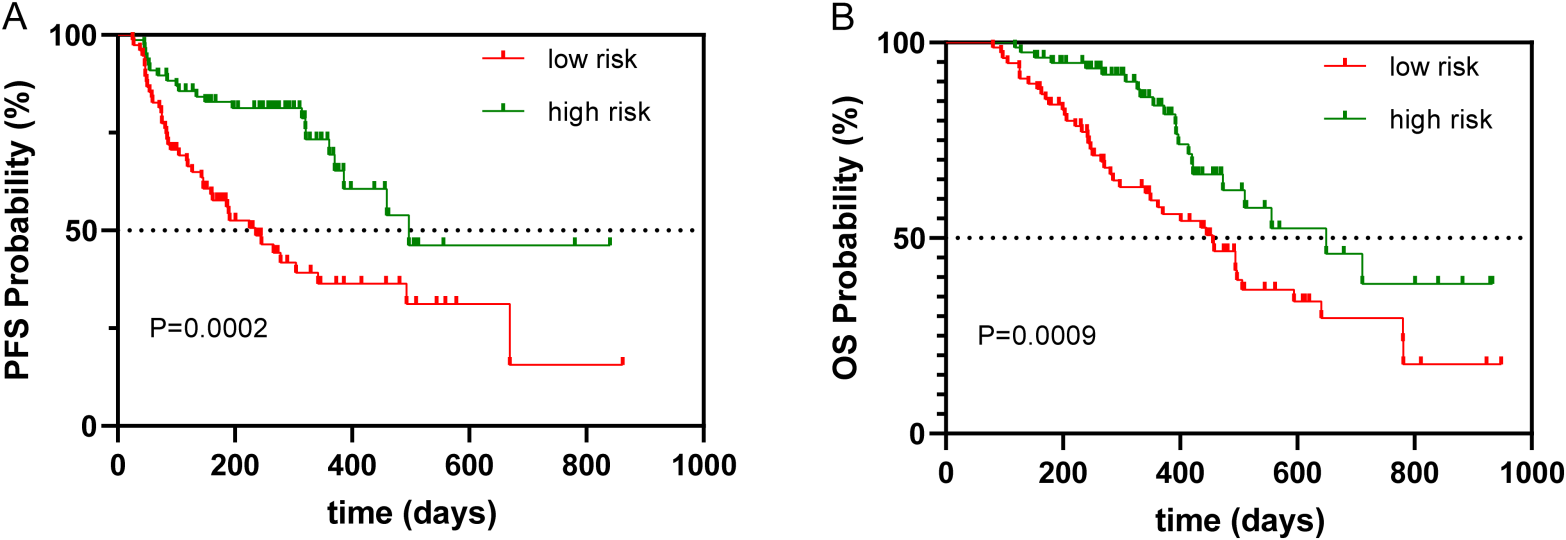
Kaplan–Meier survival curve of PNI associated with PFS and OS. **(A)** Kaplan–Meier survival curve of PNI associated with PFS, **(B)** Kaplan–Meier survival curve of PNI associated with OS.

By the study cutoff date, 112 patients (72.73%) had experienced disease progression according to RECIST criteria, while 42 (27.27%) exhibited disease stabilization or remission. During the research, 65 participants reached disease-related mortality as an endpoint event. Univariate Cox regression analysis revealed that several clinical characteristics were correlated with PFS and OS (**Table 2**). Specifically, PNI was significantly associated with both PFS (HR=0.392, 95%CI 0.234-0.657, p<0.001) and OS (HR=0.479, 95%CI 0.286-0.803, p=0.005). Consistent with this, the log-rank test and Kaplan–Meier survival curves demonstrated that patients with higher PNI levels had significantly longer PFS (**Figure 1A**) and OS (**Figure 1B**) compared to those with lower levels. Multivariate Cox regression analysis further confirmed that PNI independently influence the prognosis of PFS (HR=0.395, 95%CI 0.233-0.672, p=0.001) and OS (HR=0.515, 95%CI 0.302-0.879, p=0.015). These findings underscore the utility of PNI as a reliable predictor of PFS (**Table 3**) and OS in advanced BC (**Table 4**).

**Table 2:** Univariate cox regression analyses of variables associated with PFS and OS.

Characteristic	PFS		OS	
	HR (95% CI)	p-value*	HR (95% CI)	p-value*
Age (years)^1^				
<40.00				
≥40.00	1.441 (0.657-1.161)	0.362	1.837 (0.876-3.853)	0.108
ER				
Negative				
Positive	1.450 (0.886-2.373)	0.140	1.240 (0.759-2.026)	0.390
PR				
Negative				
Positive	1.446 (0.864-2.418)	0.160	1.170 (0.698-1.960)	0.552
Her2				
Negative				
Positive	1.125 (0.484-2.614)	0.785	0.886 (0.381-2.058)	0.778
Bone Metastasis				
Without				
With	2.320 (1.421-3.787)	**0.001**	2.225 (1.363-3.631)	**0.001**
Lung Metastasis				
Without				
With	1.813 (1.007-3.053)	**0.025**	1.752 (1.047-2.932)	**0.033**
Liver Metastasis				
Without				
With	3.839 (2.230-6.607)	**<0.001**	2.492 (1.478-4.199)	**0.001**
Brain Metastasis				
Without				
With	3.671 (1.726-7.807)	**0.001**	1.535 (0.727-3.243)	0.261
Combination therapy				
Without				
With	1.076 (0.259-4.478)	0.920	0.966 (0.234-3.984)	0.962
NLR^2^				
<2.7				
≥2.7	0.759 (0.448-1.286)	0.305	1.324 (0.812-2.159)	0.260
PLR				
<99.3				
≥99.3	0.458 (0.232-0.902)	**0.024**	0.586 (0.298-1.155)	0.123
MLR				
<0.3				
≥0.3	1.740 (1.057-2.866)	**0.029**	1.656 (1.007-2.723)	**0.047**
PNI				
<47.5				
≥47.5	0.392 (0.234-0.657)	**<0.001**	0.479 (0.286-0.803)	**0.005**

^1^Age group for PFS was low risk group (<39.00) and high risk group (≥39.00).

^2^ NLR group for PFS was low risk group (<1.80) and high risk group (≥1.80).

* The value of P<0.05 in the tables is displayed in bold.

**Table 3:** Multivariate Cox regression analyses of variables associated with PFS.

Characteristic	B	SE	HR (95% CI)	p-value
PNI				
<47.5				
≥47.5	-0.928	0.271	0.395 (0.233-0.672)	0.001
Liver metastasis				
Without				
With	1.303	0.284	3.682 (2.109-6.428)	<0.001
PLR				
<99.3				
≥99.3	-1.143	0.355	0.319 (0.159-0.639)	0.001

**Table 4:** Multivariate Cox regression analyses of variables associated with OS.

Characteristic	B	SE	HR (95% CI)	p-value
PNI				
<47.5				
≥47.5	-0.633	0.272	0.515 (0.302-0.879)	0.015
Liver metastasis				
Without				
With	1.078	0.312	2.940 (1.594-5.421)	0.001
PLR				
<99.3				
≥99.3	-0.858	0.382	0.424 (0.200-0.897)	0.025
Bone metastasis				
Without				
With	0.560	0.260	1.751 (1.051-2.915)	0.031
Age (years)				
<40.00				
≥40.00	1.202	0.429	3.328 (1.435-7.717)	0.005
PR				
Negative				
Positive	-0.743	0.325	0.476 (0.252-0.899)	0.022

### 3.4 Evaluating and visualizing the predictive model

**Figure 2:**
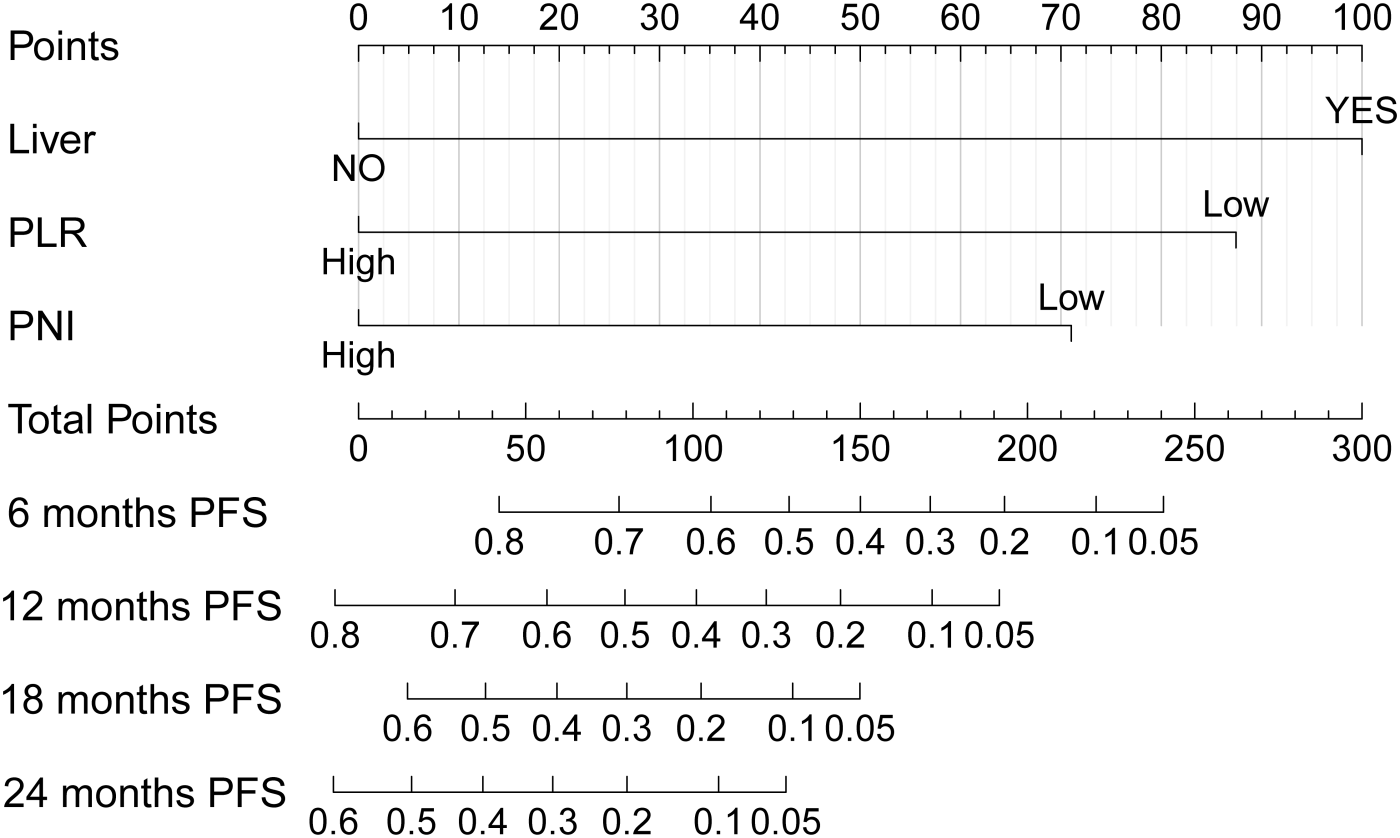
Nomogram for predicting progression-free survival (PFS) probabilities.

**Figure 3:**
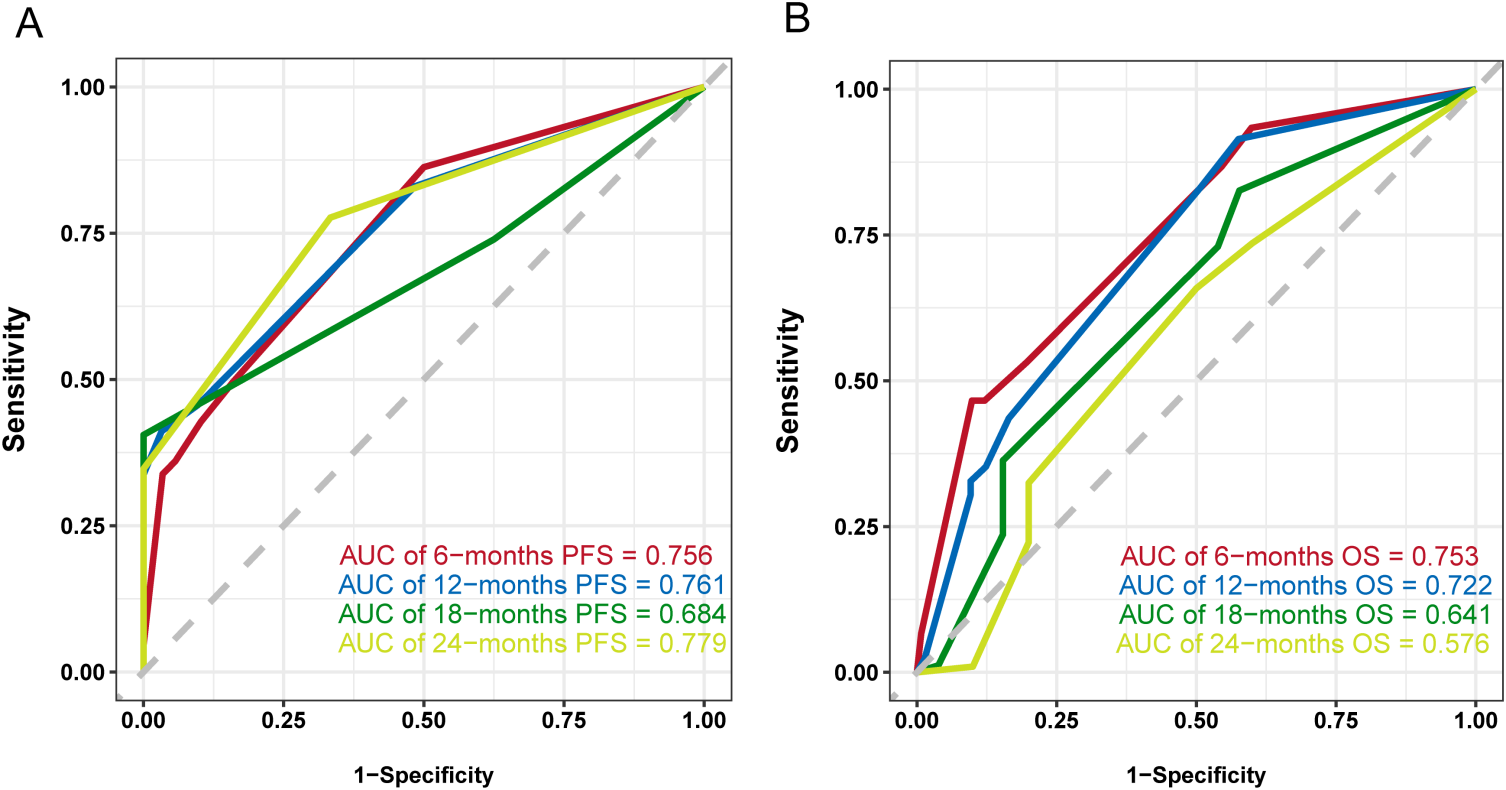
ROC curves of the nomogram model. **(A)** ROC curve and AUCs of the PFS nomogram model, **(B)** ROC curve and AUCs of the OS nomogram model.

Common independent risk factors for PFS and OS were identified through multivariate COX regression analysis. Using the *rms* package in R, nomogram prediction models for PFS (**Figure 2**) and OS (**Supplementary Figure S2**) were constructed, incorporating PNI as a key variable. The nomogram showed strong predictive capabilities, with area under the ROC (AUC) values for 6-, 12-, 18-, and 24-month PFS of 0.756, 0.761, 0.684, and 0.779 (**Figure 3A**), respectively. Similarly, the AUC values for OS at the same time points were 0.753, 0.772, 0.641, and 0.576 (**Figure 3B**). For PFS, the C-index of the nomogram was 0.710, and for OS, it was 0.705, as validated by 1,000 bootstrap resamples. Furthermore, calibration curves for 6-, 12-, and 18-month PFS (**Supplementary Figures S3A, S3C**, and **S3E**) and OS (**Supplementary Figures S3B, S3D**, and **S3F**) exhibited a strong correlation between predicted and observed survival outcomes. The clinical utility of the nomogram was affirmed by DCA, which showed it accurately predicts PFS (**Supplementary Figure S4A**) and OS (**Supplementary Figure S4B**).

## 4 Discussion

Our study proposes a novel predictive model incorporating the PNI, which demonstrates excellent predictive performance. The model was internally validated using multiple metrics, including C-index, ROC curves, calibration curves, and DCA. The nomogram further highlights the significant prognostic value of PNI in predicting clinical outcomes.

The PNI calculation relies on ALB and lymphocyte levels. ALB serves as a common measure for determining nutritional status. Many studies have shown that low ALB levels are an independent indicator of poor survival in various cancers (22). Liu et al (23). Conducted a retrospective analysis on 2,425 white female patients with non-metastatic invasive BC (stage I-III), revealing that low ALB levels were a prognostic factor for reduced survival, independent of cancer stage. Inflammation plays a critical role in all stages of tumorigenesis, including initiation, promotion, and metastatic progression (24). Inflammatory cells, like neutrophils, lymphocytes, monocytes and platelets, are integral to these processes. Lymphocytes, in particular, are crucial for immune defense, surveillance, and anti-tumor responses. Scores derived from lymphocytes have been proven to have prognostic value in BC, with studies showing that NLR (25), MLR (26), and PLR (27) are significantly associated with breast cancer prognosis. PNI is closely related to the development of tumors. Fu et al (28). showed that lymphocytes and fibroblasts infiltrate more densely in the tumor microenvironment of the high PNI group, suggesting a stronger anti-tumor immune response. Choi et al (29). further showed that the density of CD4^+^T cells in the tumor microenvironment of patients in the low PNI group, was significantly reduced, and CD4^+^T cells exert anti-tumor effects by activating CD8^+^T cells. Therefore, low PNI may lead to immunosuppression and promote tumor progression. Chen et al (30). analyzed 785 breast cancer patients and identified a PNI cutoff value of 51 based on the ROC analysis, finding that patients with a higher PNI, accompanied by longer disease-free survival (DFS) and OS, had significant outcomes (P<0.001). In addition, PNI is often used in combination with inflammatory markers (such as NLR, PLR, CRP) to form a more comprehensive prognostic model. Our approach involved using recognized statistical methods, such as Cox regression analysis and Kaplan–Meier survival analysis, to assess the connection between PNI levels and patient outcomes like PFS and OS. The findings revealed that PNI was a significant predictor (p < 0.05), highlighting its usefulness in categorizing patients according to prognosis. Based on Multivariate analyses, this study constructed a nomogram model including PNI. Clinicians can calculate patient risk scores based on the model and estimate long-term survival rates, so as to better identify advanced BC patients who can benefit from immunotherapy and guide clinical individualized treatment.

This research, however, is not without its limitations. Initially, the study was conducted retrospectively and only involved Chinese subjects, which fails to entirely eliminate the possibility of selection bias and limited the generalizability of findings across different ethnic groups. Multi-center prospective research is needed to further verify in the future. Second, the research was constrained by a relatively small participant pool and insufficient follow-up duration, necessitating further investigation with an expanded sample size and extended follow-up periods. Furthermore, this study included only the baseline data of laboratory indicators, did not dynamically monitor the changes during treatment, and only conducted internal verification and no external verification, so relevant analysis is required. Despite these limitations, the findings also provide valuable insights for the clinical application of immunotherapy.

## 5 Conclusion

In this research, PNI was determined to be an independent prognostic factor for patients with advanced BC receiving immunotherapy. Patients with higher PNI levels exhibited significantly longer PFS and OS compared to those with lower levels. Furthermore, a predictive model was constructed based on PNI, with the resulting nomogram exhibiting satisfactory predictive ability, suggesting its potential as a clinical tool for estimating PFS and OS in this patient population.

## Data Availability

The original contributions presented in the study are included in the article/**Supplementary Material**. Further inquiries can be directed to the corresponding authors.

## Supplementary material

Supplementary Figure S1 The flowchart presented the screening process for eligible patients.

Supplementary Figure S2 Nomogram for predicting overall survival (OS) probabilities.

Supplementary Figure S3 Calibration curves of the nomogram model. **(A)** Calibration curve of 6-months PFS, **(B)** Calibration curve of 6-months OS, **(C)** Calibration curve of 12-months PFS, **(D)** Calibration curve of 12-months OS, **(E)** Calibration curve of 618-months PFS, **(F)** Calibration curve of 18-months OS.

Supplementary Figure S4 DCA curves of the nomogram model. **(A)** DCA curve of the PFS nomogram model, **(B)** DCA curve of the OS nomogram model.
