# Correction: PNI as a predictive biomarker: a novel nomogram of immunotherapy efficacy in advanced breast cancer

**DOI:** 10.3389/fonc.2025.1694570

**Published:** 2025-09-11

**Authors:** Xinyang Li, Xiang Cheng, Yikai Han, Xiaodan Liu, Yujin Fang, Shengju Ren, Xiangwen Dong, Ziwen Lei, Yue Zhang, Tengfei Zhang

**Affiliations:** ^1^ The First Affiliated Hospital of Henan University of Chinese Medicine, Zhengzhou, Henan, China; ^2^ Department of Oncology, The First Affiliated Hospital of Zhengzhou University, Zhengzhou, Henan, China; ^3^ Department of Oncology, Dengzhou Central Hospital, Dengzhou, Henan, China; ^4^ Medical School, Huanghe Science and Technology University, Zhengzhou, Henan, China

**Keywords:** breast cancer, nomogram, prognostic nutritional index, immunotherapy, prognosis

Affiliation “The First Affiliated Hospital of Henan University of Chinese Medicine, Zhengzhou, Henan, China” was omitted from the paper. This affiliation has now been added for author Tengfei Zhang and Xinyang Li.

The corresponding author list appeared in the wrong order. The correct version is "Tengfei Zhang: fcczhangtf@zzu.edu.cn, Yue Zhang: 814031339@qq.com".

The original version of this article has been updated.

