# Supplementary figures and images for: PNI as a predictive biomarker: a novel nomogram of immunotherapy efficacy in advanced breast cancer
Source: Front Oncol. 2025 Aug 15;15:1534545. doi: 10.3389/fonc.2025.1534545 (PMC12395289; doi:10.3389/fonc.2025.1534545)

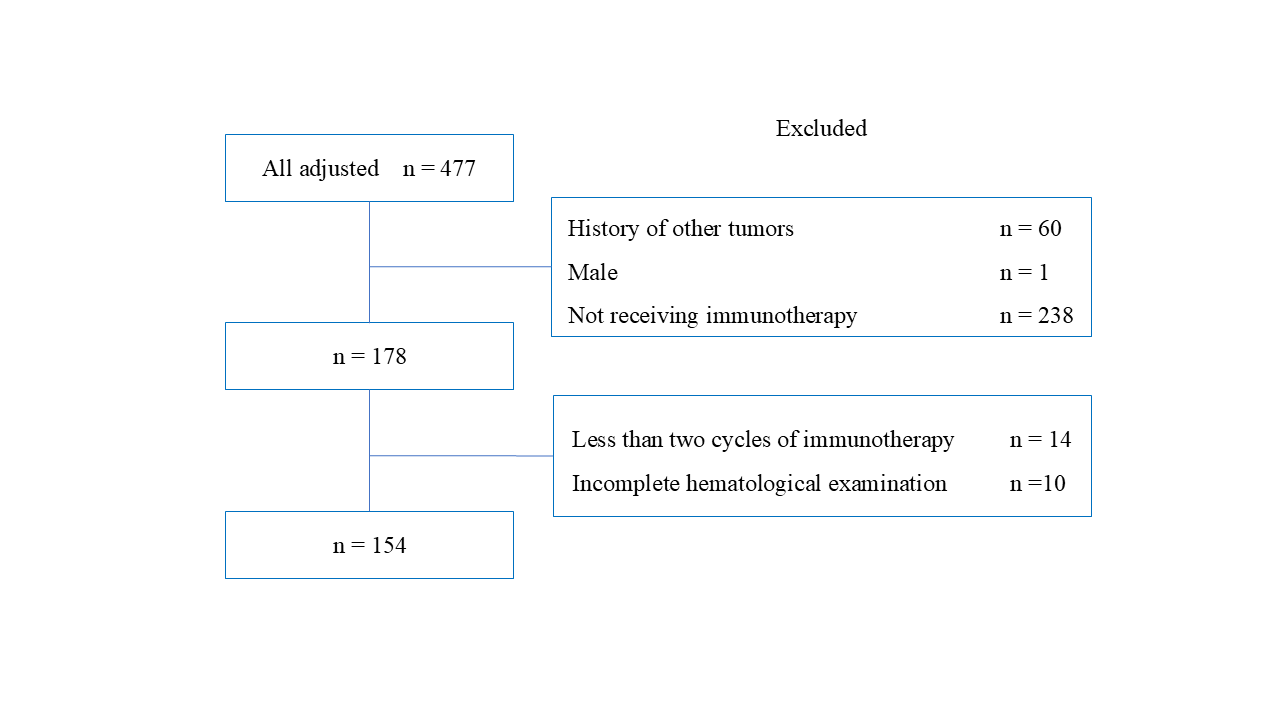

Supplement: Supplementary Figure S1 — The flowchart presented the screening process for eligible patients. [file Image1.tif]

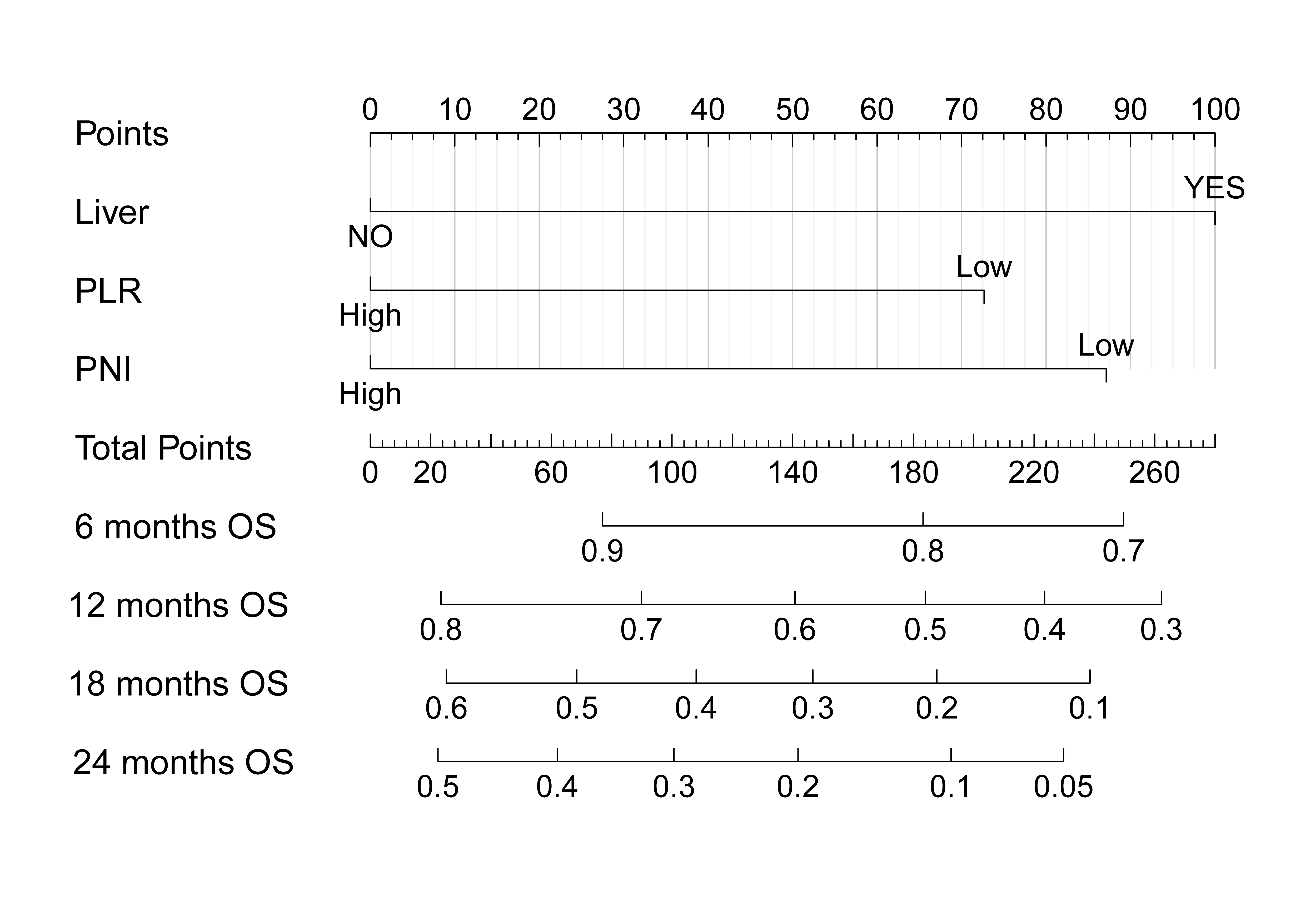

Supplement: Supplementary Figure S2 — Nomogram for predicting overall survival (OS) probabilities. [file Image2.tif]

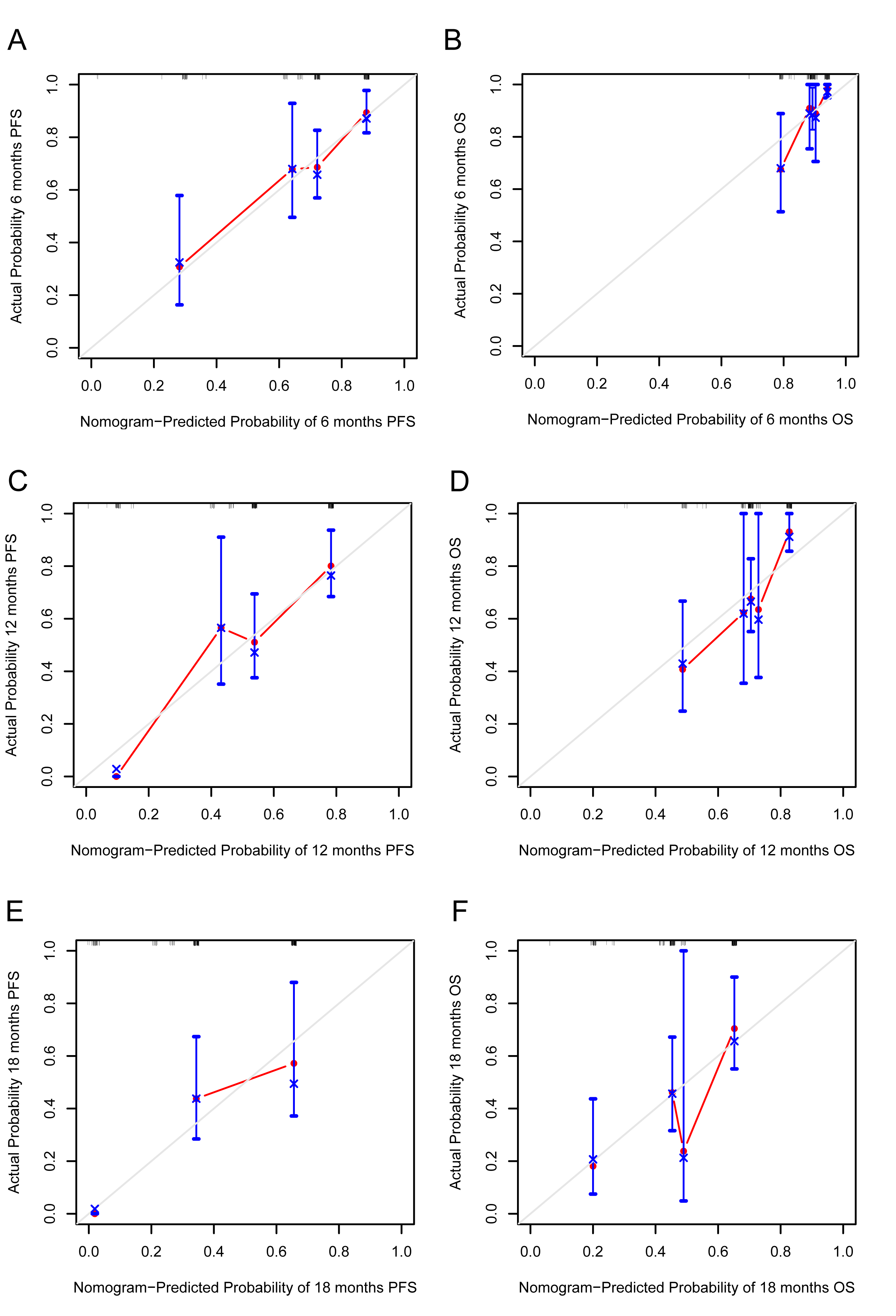

Supplement: Supplementary Figure S3 — Calibration curves of the nomogram model. (A) Calibration curve of 6-months PFS, (B) Calibration curve of 6-months OS, (C) Calibration curve of 12-months PFS, (D) Calibration curve of 12-months OS, (E) Calibration curve of 618-months PFS, (F) Calibration curve of 18-months OS. [file Image3.tif]

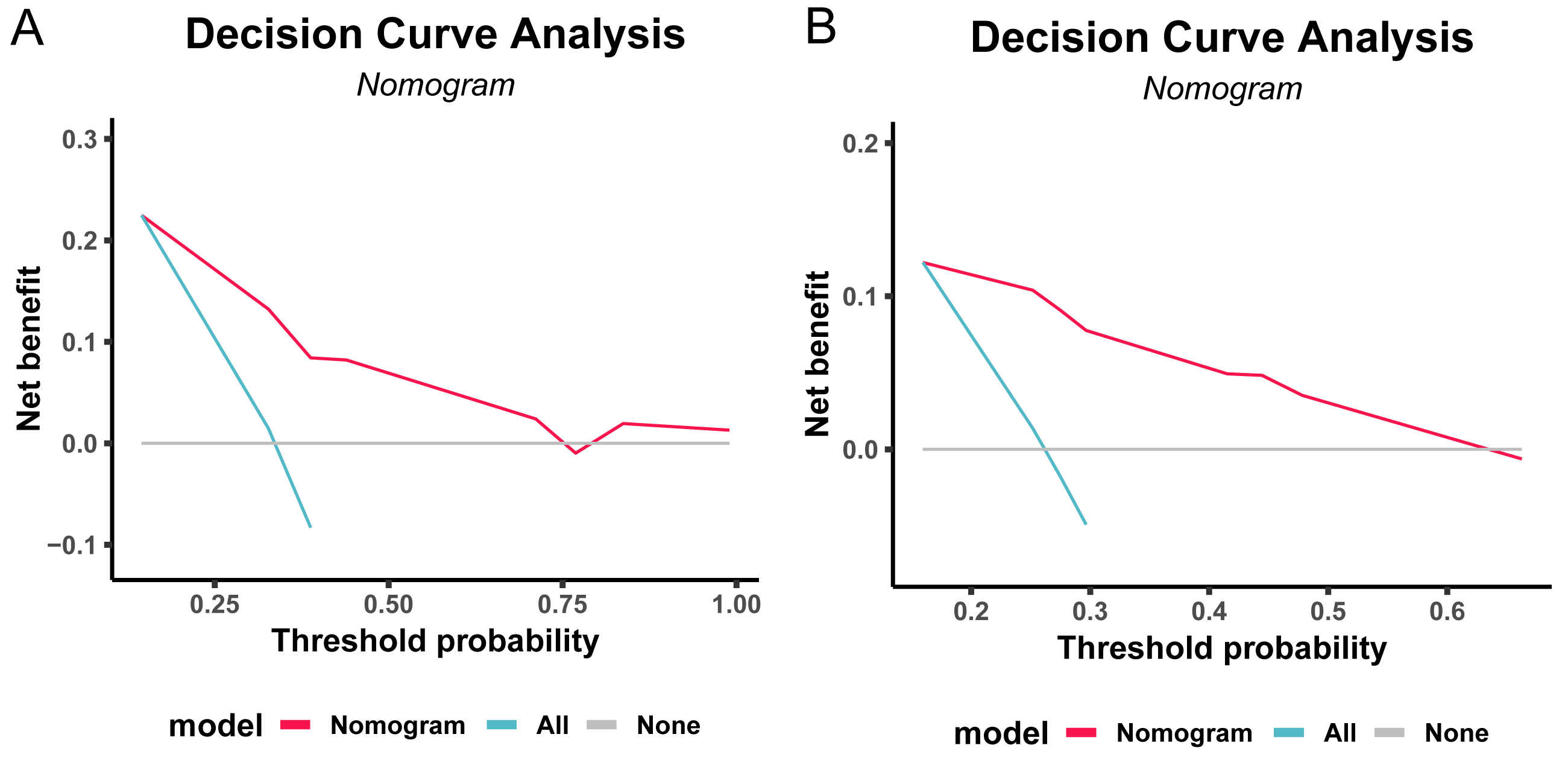

Supplement: Supplementary Figure S4 — DCA curves of the nomogram model. (A) DCA curve of the PFS nomogram model, (B) DCA curve of the OS nomogram model. [file Image4.tif]
